# Effect of the SPRING home visits intervention on early child development and growth in rural India and Pakistan: parallel cluster randomised controlled trials

**DOI:** 10.3389/fnut.2023.1155763

**Published:** 2023-06-19

**Authors:** Betty R. Kirkwood, Siham Sikander, Reetabrata Roy, Seyi Soremekun, Sunil S. Bhopal, Bilal Avan, Raghu Lingam, Lu Gram, Seeba Amenga-Etego, Bushra Khan, Sarmad Aziz, Divya Kumar, Deepali Verma, Kamal Kant Sharma, Satya Narayan Panchal, Shamsa Zafar, Jolene Skordis, Neha Batura, Assad Hafeez, Zelee Hill, Gauri Divan, Atif Rahman

**Affiliations:** ^1^Department of Population Health, Faculty of Epidemiology & Population Health, London School of Hygiene & Tropical Medicine, London, United Kingdom; ^2^Department of Primary Care and Mental Health, University of Liverpool, Liverpool, United Kingdom; ^3^Global Institute of Human Development, Shifa Tameer-e-Millat University, Islamabad, Pakistan; ^4^Child Development Group, Sangath, New Delhi, India; ^5^Department of Infection Biology, Faculty of Clinical Research, London School of Hygiene and Tropical Medicine, London, United Kingdom; ^6^Population Health Sciences Institute, Newcastle University, Newcastle upon Tyne, United Kingdom; ^7^Population Child Health Research Group, School of Clinical Medicine, University of New South Wales, Randwick, NSW, Australia; ^8^Institute for Global Health, University College London, London, United Kingdom; ^9^Kintampo Health Research Centre, Ghana Health Service, Kintampo, Ghana; ^10^Department of Psychology, University of Karachi, Karachi, Sindh, Pakistan; ^11^Department of Anthropology, University College London, London, United Kingdom; ^12^Fazaia Medical College, Air University, Islamabad, Pakistan; ^13^World Health Organization, Kuwait City, Kuwait

**Keywords:** early child development (ECD), child growth and nutrition, home visits, nurturing care, cluster randomised control trial, community health worker (CHW), India, Pakistan

## Abstract

**Introduction:**

Almost 250 million children fail to achieve their full growth or developmental potential, trapping them in a cycle of continuing disadvantage. Strong evidence exists that parent-focussed face to face interventions can improve developmental outcomes; the challenge is delivering these on a wide scale. SPRING (Sustainable Programme Incorporating Nutrition and Games) aimed to address this by developing a feasible affordable programme of monthly home visits by community-based workers (CWs) and testing two different delivery models at scale in a programmatic setting. In Pakistan, SPRING was embedded into existing monthly home visits of Lady Health Workers (LHWs). In India, it was delivered by a civil society/non-governmental organisation (CSO/NGO) that trained a new cadre of CWs.

**Methods:**

The SPRING interventions were evaluated through parallel cluster randomised trials. In Pakistan, clusters were 20 Union Councils (UCs), and in India, the catchment areas of 24 health sub-centres. Trial participants were mother-baby dyads of live born babies recruited through surveillance systems of 2 monthly home visits. Primary outcomes were BSID-III composite scores for psychomotor, cognitive and language development plus height for age *z*-score (HAZ), assessed at 18 months of age. Analyses were by intention to treat.

**Results:**

1,443 children in India were assessed at age 18 months and 1,016 in Pakistan. There was no impact in either setting on ECD outcomes or growth. The percentage of children in the SPRING intervention group who were receiving diets at 12 months of age that met the WHO minimum acceptable criteria was 35% higher in India (95% CI: 4–75%, *p* = 0.023) and 45% higher in Pakistan (95% CI: 15–83%, *p* = 0.002) compared to children in the control groups.

**Discussion:**

The lack of impact is explained by shortcomings in implementation factors. Important lessons were learnt. Integrating additional tasks into the already overloaded workload of CWs is unlikely to be successful without additional resources and re-organisation of their goals to include the new tasks. The NGO model is the most likely for scale-up as few countries have established infrastructures like the LHW programme. It will require careful attention to the establishment of strong administrative and management systems to support its implementation.

## Introduction

Early childhood development (ECD) has risen exponentially in policy importance over the last 10 years (1). It is embedded in several of the Sustainable Development Goals (2), is explicit in the new vision of the UN Secretary-General’s Global Strategy for Women’s, Children’s and Adolescents’ Health 2016–2030 with the objectives of Survive, Thrive and Transform (3), and has been prioritised in the work programmes of several global institutions, including UNICEF, the World Bank, UNESCO and the World Health Organization (4). This culminated in the launch of the Nurturing Care Framework (NCF) for ECD during the 71st World Health Assembly in May 2018 (5), where it was argued that “Investing in ECD is one of the best investments a country can make to boost economic growth, promote peaceful and sustainable societies, and eliminate extreme poverty and inequality.” The NCF and this statement are underpinned by considerable advancements in science over the last 30 years (6–10).

The Lancet Series on ECD estimated that almost 250 million children under the age of 5 years who live in low- and middle-income countries (LMICs) are at high risk of not reaching their developmental potential (11). Many are likely to do poorly in school and subsequently as adults many will have poor health, high fertility, and provide poor health care, nutrition, and stimulation to their own children, thus contributing to the intergenerational transmission of disadvantage (12). The loss of human potential that this represents is also associated with more than a 20% deficit in adult income and will have negative implications for national development (13, 14).

The NCF is a road map for action built around five components: good health; adequate nutrition; responsive caregiving; security and safety; and opportunities for early learning. It focusses on the critical period from pregnancy to age 3, during which neuroplasticity is at its peak with new neural synaptic connections made in response to interactions with the environment, providing the foundation for healthy physical and mental health throughout the life course (6).

Strong evidence exists from several randomised controlled trials that parent-focussed face to face interventions can improve child development outcomes and can allow children to compensate for delays in their development due to diverse risks, such as malnutrition and poverty (15–17). This evidence is summarised in a World Health Organization ECD guideline which recommends supporting responsive care and early learning activities for all young children, providing such support in conjunction with nutrition interventions, and promoting maternal mental health. The guideline outlines that the challenge now is understanding how to deliver such interventions effectively at scale across diverse contexts (5, 18).

The Wellcome Trust SPRING programme in India and Pakistan was designed to address this challenge. Both countries featured in the 2016 Lancet ECD Series list of the top 10 countries in 2010 with the largest number of children at risk of impaired cognitive and social–emotional development, as a result of stunting or extreme poverty (11). These countries were, in order of numbers affected, India, China, Nigeria, Bangladesh, Indonesia, Pakistan, Ethiopia, DR Congo, Tanzania, and the Philippines. The 2021 UNICEF ECD country profiles give 2015 estimates of 45% for India and 54% for Pakistan (19), while the WHO 2022 World Health Statistics report give estimates of the percentage of children stunted as 30.9% for India and 36.7% for Pakistan, both estimates from 2020 (20).

SPRING stands for Sustainable Programme Incorporating Nutrition and Games. The aim was to develop an integrated nutrition and child development intervention package to support families to give their children the best start in life, that was designed from the outset to be feasible, affordable and appropriate for delivery at scale, and to test this through cluster randomised controlled trials in two settings, using two different delivery models for scale-up using community-based workers (CWs). In Pakistan, the SPRING intervention was embedded into the existing monthly home visits of the Lady Health Workers (LHWs). In India, home visits were implemented by a civil society/non-governmental organisation (CSO/NGO) that trained a new cadre of CWs, called Kilkaari Workers (KWs) to work alongside the existing maternal and child health community services provided by ASHAs and Anganwadi workers (21).

The primary objectives were: (1) To design the SPRING integrated nutrition and child development intervention, based on extensive formative research and best practise guidelines; (2) To test the SPRING intervention through parallel cluster randomised controlled trials in India and Pakistan; (3) To evaluate the impact of SPRING on early child growth and development, and on intermediate outcomes that lie on the hypothesised change pathway including quality of the home environment, infant feeding practises and maternal psychosocial distress; (4) To evaluate and monitor all aspects of the SPRING intervention process and implementation; and (5) To cost the delivery of the SPRING intervention and assess its cost effectiveness.

In this paper we present the impact of SPRING on early child development and growth in the first 18 months of life. A companion paper in this supplement presents findings from the process evaluation together with lessons learned for delivering community based ECD interventions at scale (22).

## Methods

### Trial design

The SPRING intervention was evaluated through parallel cluster randomised trials carried out in Rawalpindi District in Pakistan and Rewari district in the south of Haryana in India. Clusters were chosen to be supervisory zones of the CWs to minimise any chance of contamination. In Pakistan, clusters were 20 Union Councils (UCs), which cover populations of about 22,000 and which are the supervisory zones of the LHWs. There are approximately 15–20 LHWs in a UC. Each LHW is a local resident of the community she serves, covering a population of 1,200–1700 and approximately 250–275 households. They are paid by the government, have a minimum 8–10 years of education, and are trained for 15 months to carry out monthly home visits to provide preventive maternal and child health services along with other duties (23). Each UC has one LHW supervisor (LHS) responsible for holding monthly group supervisory meetings and carrying out one to one field supervision of LHWs. LHWs in 10 of the UCs chosen at random were trained to deliver the SPRING intervention alongside their other activities.

In India, clusters were the catchment areas of 24 health sub-centres with functional Auxiliary Nurse Midwives and covering a population of at least 8,000. They were the supervisory zones of the ASHAs, a comparable cadre to the project appointed Kilkaari Workers (KWs) who were recruited in 12 of the clusters chosen at random to deliver the SPRING intervention through monthly home visits. Each intervention cluster had 3–6 KWs each serving a maximum of a hundred homes with eligible families. If the potential workload in a single cluster did not justify a full-time appointment the KW covered two clusters. KWs were recruited by the implementing NGO using similar criteria as those used for ASHA workers in that they were: Married, educated till 8th grade or above, lived in the community they served – or in the adjacent community, and had good communication skills.

### Surveillance system

The trials were supported by surveillance systems of regular 2-monthly visits by trained fieldworkers to identify pregnant women and newborns. There was one resident fieldworker (FW) in each cluster, making a total of 24 in India and 20 in Pakistan with one field supervisor per 5 FWs and a head of FW in each site. Training consisted of 3 phases of 3–5 days length covering: mapping of the study area; baseline survey; and the two-monthly surveillance visits. In India the surveillance system covered all households in the trial clusters. In Pakistan the surveillance system covered all households within sub-areas designated as evaluation zones within each cluster (Union Councils). These evaluation zones were chosen to be comparable in population size to the clusters in India and comprised 7 LHW catchment areas, out of the 15 LHW areas in each Union Council. Each zone was defined by selecting one LHW area at random and then adding the 6 nearest LHW areas to form a contiguous evaluation zone. This selection was carried out by the SPRING statistician who had no knowledge of the areas.

All women of reproductive age were enrolled into the surveillance system at the first household visit if they were aged less than 50 years, married, not sterilised and whose husbands were not sterilised. Additional women were enrolled at subsequent visits if they met the criteria, for example if a woman became married or if an eligible woman moved into a cluster. Visits took place every 8 weeks in India and every 10 weeks in Pakistan.

### Participants

Mother-baby dyads of all live born babies identified by the surveillance system in trial clusters who were born on or after the date of full implementation of SPRING were enrolled into the SPRING trial. This was 2 May 2014 in Pakistan and 18 June 2015 in India allowing for a 2–3 month embedding period. Exclusion criteria were babies with major congenital malformations, babies whose mothers died and mothers who were incapable of answering questions. Socio-economic and birth data were collected at enrolment. Data on contact with maternal and child health and community workers and on breastfeeding and complementary feeding practises were collected at all subsequent home visits up to 30 April 2016 in Pakistan and 01 July 2017 in India, when primary outcome data collection was completed.

The first mother-baby dyads identified were recruited in the child development assessment (CDA) subsample for assessments of primary outcomes when children reached 12 months of age and again at 18 months of age; these assessments were carried out by specialist outcome assessment teams. CDA recruitment continued until sample size requirements were met. Additional mothers were recruited for assessment of maternal mental and social wellbeing, family support and maternal efficacy at the 12-month assessment as these intermediate outcomes required a larger sample size; they are not included in this paper.

### Interventions

#### Spring intervention

In India, SPRING was branded “Kilkaari,” the happy gurgling of a small child, and delivered by CWs called Kilkaari workers (KWs), a new cadre of CWs, specifically recruited by SANGATH, the implementing NGO, to only deliver the SPRING intervention. They were recruited to have characteristics similar to government frontline workers, and received similar renumeration. KWs were resident in the community, had a minimum of 8th grade education, were married and had good communication skills. Each served a maximum of 100 eligible households. They were asked to engage with the existing maternal and child health community services provided by ASHAs and Anganwadi workers and attend local community events such as Village Health Days to identify new pregnancies and mothers with young children.

In Pakistan, SPRING was branded “Roshan Kal,” a bright tomorrow. It was embedded in the government Lady Health Worker (LHW) monthly home visit programme which was established in 1994 (23). The LHW role comprises over 20 maternal and child health services including health education on breastfeeding and complementary feeding, child growth monitoring, immunisations, family planning and basic curative care. Within the government programme LHWs receive 15 months training, are aged 18–45 years, resident in the community they serve, have a minimum 8 years of education, are preferably married and are acceptable to the community. Each Union Council has 15–20 LHWs, each serving 150–200 households. LHWs conduct approximately 7 home visits per day and the SPRING content was integrated into these home visits.

**Figure 1:**
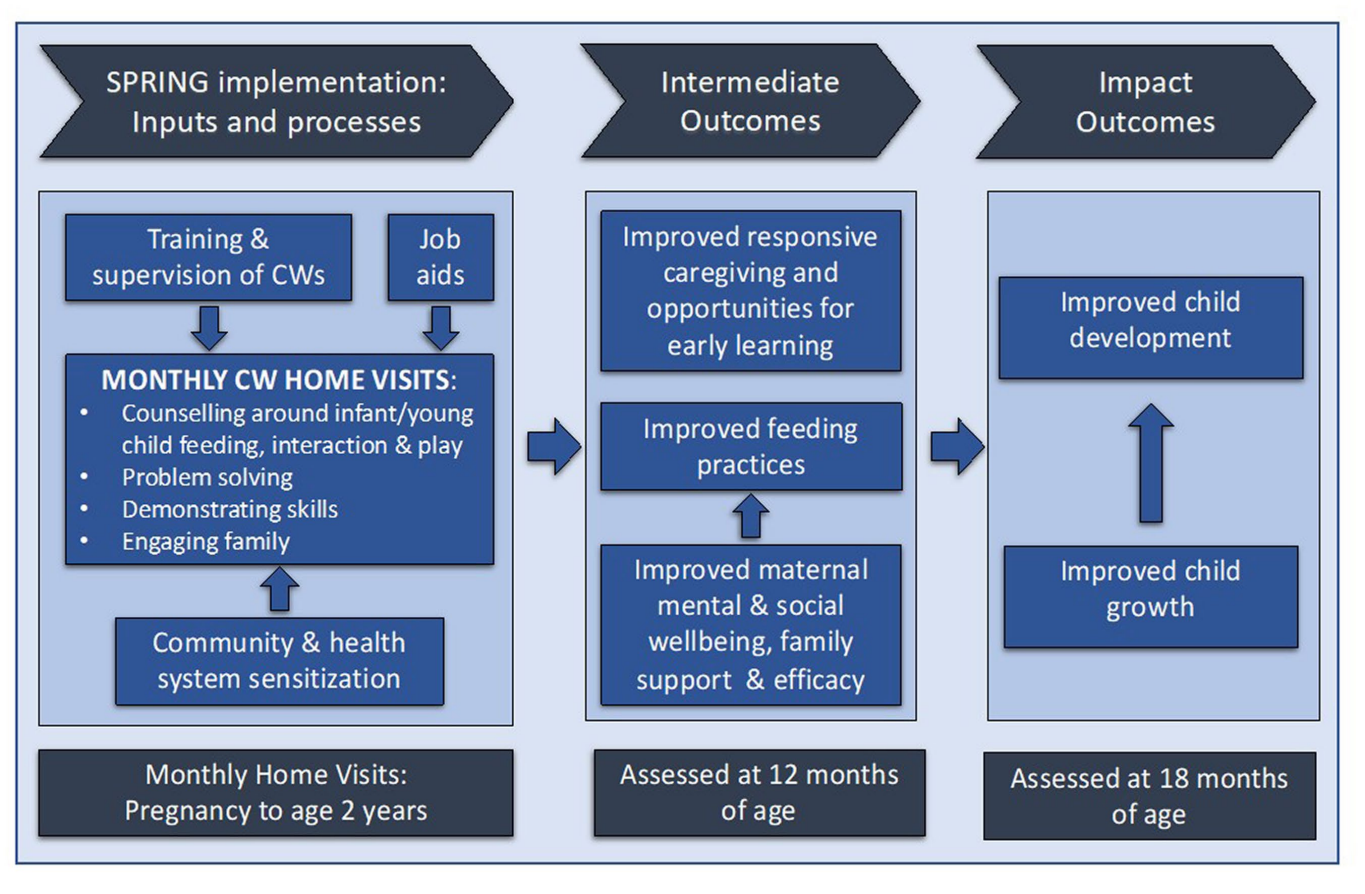
SPRING conceptual framework and change pathway.

The SPRING intervention targeted infant/young child feeding and interaction and play with the aim of improving child growth and development. It was guided by the conceptual framework & change pathway shown in Figure 1, which was used both during the development of the intervention and to guide the intermediate outcome data collected in the evaluation. The content was based on formative research into existing behaviours, and into barriers to and facilitators for adopting desired ECD and feeding practises (24). The desired behaviours for play were adapted from the WHO/UNICEF Care for Development curriculum (25), and those for complementary feeding from WHO/UNICEF infant and young child feeding guidelines (26).

It was delivered through monthly home visits by the CWs to mothers starting in pregnancy and continuing through the first 2 years of a child’s life (Table 1). Home visits in pregnancy focused on maternal health and sensitisation about breastfeeding. Postnatal visits focused on breastfeeding, complementary and responsive feeding, and play activities, with new messages introduced each month dependent on the age if the child. The CWs were trained to engage with and include other family members in the visits as appropriate.

**Table 1:** SPRING intervention – content of CW monthly home visits.

Age at visit	Nutrition related	Play and stimulation
Pregnancy	Importance of family support for maternal and child well-being	
	Encouragement of family involvement	
	Iron during pregnancy	
	Diet and rest during pregnancy	
	Early and responsive breastfeeding with love and care	
	No pre-lacteal feeds	
Neonatal period	Exclusive and responsive breastfeeding with love and care	Talking or singing to the child while breastfeeding and doing daily activities, looking into the child’s eyes during breastfeeding and allowing freedom of movement of limbs
	Avoiding insufficient milk through diet and frequent breastfeeding	
1–11 months	Continue exclusive breastfeeding until 6 months and then continued breastfeeding	Child and age specific activities such as: following objects, grabbing objects, copying sounds and actions, clapping, saying bye bye.
	In last visit before 6 months, introduce concept of weaning from 6 months	
	Complementary feeding age dependent messages on: Responsive feeding Consistency Frequency, variety and quantity Hygiene Adding “super” foods (eg butter or oil) Finger food Enjoyable meal times Avoiding “junk” foods (Pakistan only)	
	Importance of feeding for a sick child	
Second year	Eating with the family and balanced diet	Child and age specific activities such as: stacking; putting in and out; naming objects; following instructions; colour matching.
	Continued breastfeeding	

The CWs used a counselling approach based on cognitive behaviour therapy (CBT) that had successfully been used with LHWs in a previous study in Pakistan (27). The approach comprises 6 key principles: family support; guided discovery using pictures; behavioural activation; empathic listening; problem-solving; and praise. The child development component included practical coaching of families on stimulation activities, where CWs explained the child stimulation activities and demonstrated if needed and then coached families on key elements such as praising the child and scaffolding new activities as appropriate. This practical component aimed to enhance caregiver self-efficacy, skills and recall. CWs were trained to use counselling cards, which included culturally appropriate illustrations for the family to look at and instructions and key messages for the CW to deliver, that were simple to use and easy to understand. In Pakistan, CWs also gave families pictorial Roshan Kal calendars, which comprised reminder messages and a space for families to tick if they had been able to follow the behaviours discussed.

##### Training

In both sites the CWs were trained by SPRING supervisors who were female social science graduates overseen by senior project staff. Training was divided into two phases of 5 and 3 days, to ensure the quantity of material covered was manageable for the CWs, and to maximise experiential learning. Maximum group size was 29. The first training phase focused on ensuring the CWs had the core knowledge and competencies required to counsel and problem solve with families, as well as covering the background to child development and the content of the home visits up to the fifth month of age. The second phase focused on the content from 5 months to 2 years of age.

##### Supervision

In Pakistan the LHWs were supervised by integrating a SPRING supervision component into the LHW routine monthly group supervision meetings with the LHS, and through SPRING specific one-to-one supervision visits in the field. Once the LHS had completed their group supervision, the SPRING supervisor led a session that focused on sharing experiences, peer-learning, problem-solving, skills development through role play, motivation and peer support. They also identified issues faced by LHWs in the field, helped develop solutions and identified further training needs. The same SPRING supervisors provided individual feedback on LHW performance during monthly observation of a home visit. The supervision in India followed the same structure but the group supervision was arranged by the SPRING team and the size of the groups was slightly smaller ranging from 11 to 16 KW workers.

During the observed home visit the supervisors completed a checklist to record visit quality for monitoring purposes and provided supportive feedback after the completion of the visit. The tone of individual supervision followed the SPRING principles of empathetic listening, praise for the CW on things they had done well, suggestions and problem solving on areas that they could improve on, and encouragement to adopt a more counselling-based approach if needed.

##### Process evaluation

Quantitative process data were collected on training (self-completed pre and post training test), supervision coverage (programme records), visit coverage (caregiver interviews) and visit quality (field supervision checklist and caregiver interviews). Qualitative data on acceptability and barriers and facilitators for change were collected through in-depth interviews with mothers (24); focus group discussions with mothers (8), grandmothers (12), and fathers (12) and in-depth interviews or focus group discussions with the community-based agents and their supervisors (13) provided. These data were not used to course correct in order to improve the quality of intervention delivery during the trial. The supportive supervision with its emphasis on problem solving was the only strategy used to do this.

#### Control interventions

Pregnant women and newborn babies living in both intervention and control zones continued to benefit from the routine maternal and child health care available. This consisted of home-visiting advice by LHWs in Pakistan, and home- and centre-based advice and care from ASHAs, Anganwadi Workers, and Auxillary Nurse Midwives in India, plus access to routine maternal- and child-healthcare services in both countries.

### Trial outcomes

#### Impact outcomes: child development and growth

Child development and growth were assessed by a team of outcome assessors (OAs) when trial children reached 18 months of age; this was changed from 24 months of age with agreement by the trial steering committee (TSC) at its meeting on 26 November 2013 due to political instability in Pakistan and a site-change in India. The time window for the assessments was −7 days to +21 days of the exact date the child reached 18 months of age. The assessments took a total of 2–3 h to carry out.

The OAs worked in pairs. They administered the gross motor, fine motor, cognitive, receptive language and expressive language subtests of the Bayley Scales of Infant and Toddler Development, Third Edition (BSID-III) (28). The subtests consist of a series of increasingly complex tests (items) covering ages 1–42 months. The assessment starting point depends on the child’s age. This was section K (age 16 m 16 days – 19 m 15 days) for SPRING trial children who were aged 18 months. Items were administered until the child was not able to complete five consecutive items in a row, at which point the assessment for the subtest ended. Children received a point for every successfully completed item from their starting point plus points for all the items in the sections before their starting point. If a child was unable to complete the first three items of section K, the OA started the assessment from the beginning of section J (age 13 m 16 days – 16 m 15 days) instead, and if still not successful from the beginning of section I (age 11 m 0 days – 13 m 15 days). If this was still not successful, the assessment was aborted; the child received only the points for the items up to the end of section H.

Three child development outcomes, mean composite psychomotor, cognitive and language development scores, were calculated from the BSID-III subtest scores as specified in the BSID-III manual (28). First each subtest score was converted to a scaled score between 1 and 19 using age-specific conversion tables in the BSID-III manual – these scaled scores allow comparability between subtest results as the number of items (and therefore maximum possible score) in the subtests varies from 48 to 91. The scaled scores on the gross and fine motor tests were then added together as were the scores for receptive and expressive language to give overall psychomotor and language scaled scores. The three domain scaled scores were then converted to composite scores, which were designed to have a mean of 100 and standard deviation of 15 in the reference population; there are two different conversion tables in the manual, one for the psychomotor and language scaled scores which have a range of 2–38, and one for the cognitive scaled score with a range of 1–19.

The OAs also weighed and measured the children. Weight was measured to the nearest 0.01Kg using SECA-384 electronic scales which were calibrated weekly. When possible, the child was weighed without their clothes. If this was not possible, the child was weighed fully clothed and the mother then asked to change the clothes and give the original clothes to the OA to be weighed; this weight was then subtracted from the weight measured. Length was measured to the nearest 0.1 cm using the SECA-417 infantometer. The mother was asked to remove the child’s shoes and lay them down on the board. One of the OAs then cupped their hands over the child’s ears and held their head against the end of the measurement board. The other then ensured that the child’s body was straight on the board, placed one hand on the child’s legs to stabilise them and brought the footpiece upwards toward the child’s feet which were held perpendicular to the board. This OA then read aloud the length board reading and this was recorded by the first assessor. Height for age, weight for age and weight for height *z*-scores were calculated using the WHO Child Growth Standards for preschool children released in 2006 (29).

All three BSID composite scores (BSID-CS) plus the height for age *z*-score (HAZ) were designated as *primary impact outcomes*. Weight for age (WAZ) and weight for height (WHZ) z-scores were designated *secondary impact outcomes*.

#### Intermediate outcomes

The two main SPRING child development and growth intermediate outcomes reflect the quality of the home environment for child development assessed using the Home Observation for Measurement of the Environment Infant-Toddler version (HOME-IT) inventory (30, 31) and the quality of infant feeding practises assessed using a questionnaire based on the WHO infant and young child feeding (IYCF) indicators (32). These were assessed within −7 days to +21 days of the date the child reached 12 months of age by OAs working in pairs. These assessments took about 2 h to complete. The intermediate outcomes are mean HOME-IT score (*primary*) and % children receiving the WHO minimum acceptable diet (*secondary*), as described in the following paragraphs. Three other *secondary* outcomes, reflecting IYCF guidelines for the first 6 months of life and based on data collected through the surveillance system, were % babies: breastfed within an hour of birth, exclusively breastfed in period 4–5 m and received solids/semisolid food at 6 months of age.

The HOME-IT inventory includes 45 items which cover six subscales: Responsivity (11 items) of the primary caregiver captures communicative and affective interactions between caregiver and child; Acceptance (8 items) assesses how caregivers discipline the child and acceptance of less than desirable behaviour; Organisation (6 items) looks at how the child’s time is organised outside the family house and what their personal space looks like; Provision of age appropriate play and Learning materials (9 items); Parental Involvement (6 items) captures how the caregivers interact physically with the child and Variety (5 items) explores how the child’s routine is designed to include social interactions with people other than the primary caregiver. Each item is scored 1 (yes) or 0 (no) based on observation or elicited through questions. An example of the former is “Mother spontaneously vocalises to the child at least twice during the interview” and an example of the latter is “Children of your child’s age can be difficult to manage. Sometimes they love to play in things that get them all messy and dirty—mud, water, their food, and so on. Is your child allowed to do this?” The number of positive responses is summed to yield a total HOME-IT score. The inventory takes about an hour to administer and to give adequate opportunity to observe mother–child interactions.

The IYCF questionnaire assesses the child’s nutritional input during the previous 24-h period described as “the whole day yesterday or last night.” Questions covered breastfeeding, ORS, medication, liquids drunk, the specific foods the child had eaten elicited through a narrative approach from the time the child woke to the time they slept, the number of meals the child had, and the mother’s estimate of the total amount eaten. The food items were categorised into 15 types in the following 6 groups: starchy food, fruit and vegetables, oil & fat and condiments, sugary foods, milk products and non-vegetarian food (eggs, fish/seafood, meet, offal), with multiple entries possible for an item. The responses were checked against the WHO criteria for the minimum acceptable diet at age 12 months defined as:

- Breastfed (bf) or had 2+ milk feeds in the last 24 h, AND
- Had food from 4 or more food groups (dairy included as food group for non-bf), AND
- Had 3 or more meals (if bf), 4 or more meals (if non-bf)

In India, the IYCF questionnaire was administered to all mothers who had a 12 m assessment and not just those whose children were recruited for the 18 m CDA.

### Cultural adaptation

All evaluation data were collected using standardised protocols by surveillance fieldworkers (FWs) and by outcome assessors (OAs), who were independent to the CWs. It was not possible to achieve blinding of cluster allocation for surveillance FWs as they were resident in the clusters. However, every effort was made to ensure blinding of the OAs who carried out assessments at 12 and 18 months of age. A systematic process of cultural adaptation was used for all instruments (33). This comprised: (a) Translation of the psychometric instruments into the local languages spoken by residents in the trial clusters, and adaptation of the testing materials for the local context; (b) Ensuring technical equivalences; (c) Cognitive interviews with respondents and project staff (field research); (d) Modifications of translated versions, based on the field research; (e) Pretesting, including further modification; (f) training of assessors including establishing an inter-rater reliability of at least 70–80%; and (g) Pilot-testing, including testing of standard operating procedures.

### Sample size

The SPRING trial aimed to recruit sufficient mother-newborn dyads to allow assessment at 18 months of age of at least 40 children in each of the 24 clusters in India and 50 in each of the 20 clusters in Pakistan, conservatively allowing for 20% loss of follow-up to 12 months of age and a further 20% follow-up loss to 18 months of age. These sample sizes were sufficient to give 90% power to detect effect sizes of 0.34SD in HAZ and 0.38SD in BSID-CSs plus 80% for gender-specific analyses. We used an estimated intra-cluster coefficient (ICC) of 0.035 for HAZ, based on the median value for rural areas reported in a review of ICCs of stunting based on height for age z-scores in DHS surveys (34). The ICC used for the BSID-CSs was 0.05; this was the ICC observed in the evaluation of the effectiveness of a parenting programme in Bangladesh to address early childhood health, growth and development, as calculated from the reported design effect (35).

### Randomisation and masking

Equal numbers of clusters in each site were randomised to intervention and control groups using baseline information collected at enrolment into the surveillance system from mothers with young children and a restricted randomisation procedure (36) to ensure balance in each site with respect to key factors related to the main trial impact outcomes. This used the following three criteria for the absolute maximum difference between intervention and control groups of: 0.5% for the percentage of children aged 18–30 months who were stunted (HAZ < -2); 2.5% for the percentage of mothers with children aged less than 5 years who had received no education; and 2.5% for the percentage of mothers with children aged less than 5 years who had delivered their youngest child in a health facility. All three measures are key determinants of child health. Stunting was chosen as the key restriction measure as it is a strong determinant of a child failing to achieve their developmental potential.

All possible allocation schemes were generated (total: 184,756 Pakistan; 2,704,156 India) and the subset meeting the criteria identified [Pakistan: 6,750 (3.7%); India: 527,776 (19.5%)]. These schemes were checked for anomalies in the frequency of co-allocations of pairs of clusters and one allocation scheme then selected at random by the trial statistician (LG) using a computer program. The allocation was shared with the SPRING implementation teams but not with the trial conduct teams.

It was not possible to achieve blinding of cluster allocation for the surveillance FWs as they were resident in the clusters. However, every effort was made to ensure blinding of the OAs who carried out assessments at 12 and 18 months of age. A strict triple blind approach was also put in place during the analysis and interpretation of the trial results. Blind analyses of trial outcomes were first discussed by the Trial Steering Committee (TSC) and Data Safety Monitoring Board (DSMB) with clusters randomly allocated to “meaningless” groups X and Y by the trial statistician using the runiform() random number generator in Stata v13. This was to agree the exact analyses to be used in reporting the trial findings and to ensure that all data procedures, methodological and statistical decisions were completed and agreed with the DSMB and TSC members before the true results were revealed, to minimise biases (37). Partially-blind analyses were then presented by groups P and Q, corresponding to intervention and control groups. The findings were then discussed, and the interpretation of any differences agreed, ignorant to the knowledge of which group was intervention and which control. A sealed envelope breaking the code was then handed to the Chair of the TSC identifying which of P and Q is the intervention group and which the control. Interpretation of findings could not be changed at this stage.

### Statistical analysis

All analyses are intention to treat and include all data from trial mothers and infants, regardless of their exposure to intervention activities. Multi-level mixed effects regression models have been used to control for the clustered nature of the data. Specifically, the cluster indicator was included as a random intercept in regression models.

Binary outcomes (e.g., whether or not an infant was receiving the WHO minimum acceptable diet) were assessed by mixed effects logistic regression, with effect sizes presented as risk ratios. These were calculated post-estimation using marginals from the logistic regression model with 95% confidence intervals (Cis) determined using the delta method (38). Continuous outcomes (child development and HOME scores, HAZ, WAZ, and WHZ) were assessed by mixed effects linear regression. The differences in mean outcomes between the two groups are also presented as effect sizes calculated as the differences divided by their pooled standard deviation; 95% CIs are given for both. Together with 95% CI.

Additional robustness check analyses were carried out to take account of factors that could have a strong relationship with the ECD and growth outcomes, and to check that no bias resulted from these. This included adjustment for month of birth, socio-economic status (SES) score (calculated using principle components analysis using data on mother, household demographics and animal and other asset ownership) and cluster level baseline factors used in the restricted randomisation (% stunting among children aged 18–30 months, % of mothers with no education among mothers with children aged less than 5 years and % of youngest children less than 5 years of age delivered in a facility). The robustness of the results from the cluster randomisation analysis strategy using random effects regression were then checked against results using randomisation inference (RI), which was run with 1,000 simulated re-randomisations of each outcome (39).

### Economic evaluation

The financial and economic costs of the SPRING intervention were estimated from the provider perspective (40, 41), using a step-down approach (42). The provider costs were those incurred in the development and implementation of the intervention. The cost data were sourced from the financial accounts of the implementing institutions and entered annually into a costing tool created in Excel. Financial costs were converted to economic costs, i.e., any donated goods or volunteer time were added to the cost sheets and assigned a current market value (43, 44). Key informant interviews with intervention staff assisted in identifying donated or subsidised items, and in allocating joint costs between programme components. The costing spanned the start-up and implementation periods. Start-up costs were differentiated from implementation costs, and research costs were not included in the analysis.

The total and average annual provider costs of the SPRING intervention package implemented in Pakistan and India were estimated. Costs were calculated in current prices in Pakistani and Indian Rupees, and converted to International Dollars to facilitate comparison with similar studies. All costs were adjusted for inflation using the Consumer Price Index for Pakistan and India, discounted at 3% per year, and converted to 2017 International Dollars (INT$) using the 2017 Purchasing Power Parity (PPP) conversion factor for both countries. The local conversion unit used to calculate 2017 INT $ for Pakistan was 28.77 and for India was 17.45 (45).

## Results

### Trial flow and recruitment

**Figure 2:**
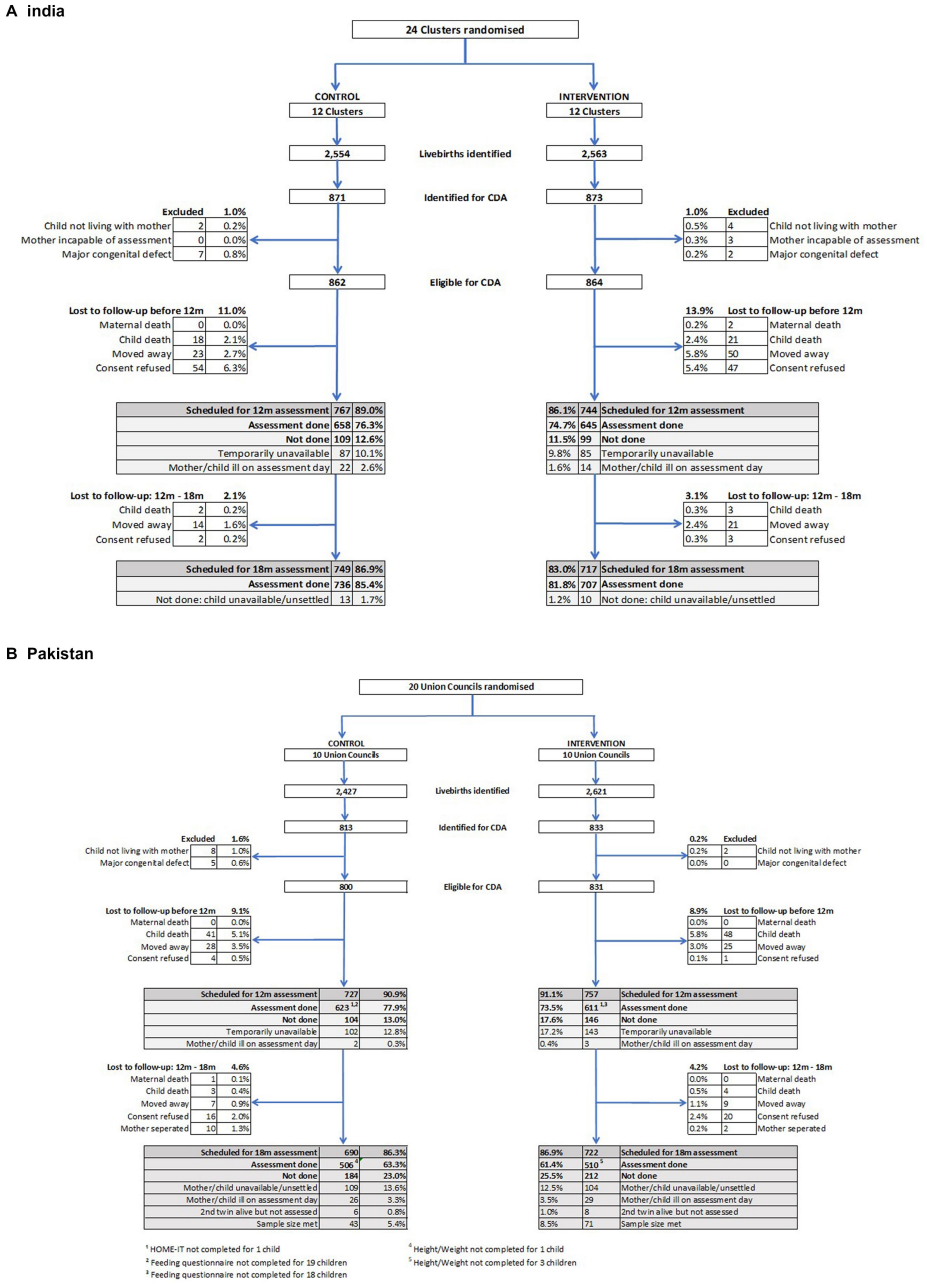
Trial profiles for babies recruited for child development assessment (CDA) at 18 months of age. **(A)** India. The 12 m feeding questionnaire was administered to all mothers who had a 12 m assessment and not just those whose children were recruited for the 18 m CDA, giving an additional 365 children in the control arm and 351 children in the intervention arm. **(B)** Pakistan.

Figure 2 shows the trial profiles for India and Pakistan. In India, 5,117 babies were identified by the surveillance system who were born alive on or after 18 June 2015, the date of full implementation of the SPRING Kilkaari intervention, with the 1744 born up to April 2016 potentially eligible for the child development assessments (CDA) at 12 and 18 months. 1726 (99%) of these babies met the eligibility criteria and were recruited into the trial. Similar numbers for Pakistan were 5,048 born on or after 2 May 2014, the date of full implementation of SPRING Roshan Kal with 1,646 identified as potentially eligible for the CDAs and 1,631 (99%) recruited. Exclusion criteria were child not living with mother at time of assessment, major congenital defect, and mother not being able to complete the assessment.

The 12-month completed assessment rates were similar in India and Pakistan, with assessments completed for 1,303 (75.5%) of the recruited mother-baby dyads in India and 1,234 (75.7%) in Pakistan. There was a 6.6% loss to follow-up in India and 9.1% in Pakistan due to maternal or child deaths or having moved away. In addition, 17.6% were lost in India and 15.3% in Pakistan due to consent refusal for the assessment or the mother/child being temporarily unavailable or ill on the scheduled assessment day. Further loss to follow-up between the 12- and 18-month assessments was 2.6% in India and 4.4% in Pakistan with a total of 1,466 children scheduled for 18-month outcome assessment in India and 1,298 in Pakistan. Of these, assessments were done with 1,443 children in India, 83.6% of those recruited, and 1,016 (62.3%) in Pakistan. The follow-up rate for the 18-month assessment in India exceeded that expected based on the allowed for losses of 20% between birth and 12 months, and a further 20% between 12 and 18 months in the sample size calculations. The follow-up rate in Pakistan was similar to that planned. Assessment rates were comparable among participants in the control and the intervention groups as can be seen in Figure 2. In India, the percentage assessed at 18 months was 85.4% in the control and 81.8% in the intervention group, a difference of −3.6% (95% CI –8.31, 1.08; *p* = 0.131). In Pakistan, the percentage was 64.1% in the control and 61.9% in the intervention group, a difference of −2.2% (−6.8, 2.5; *p* = 0.368).

### Comparability between intervention groups

There was excellent comparability at recruitment between intervention and control groups arms with respect to key characteristics for babies who were assessed at 18 months for impact outcomes (Table 2). There was also good comparability between those who were assessed and those who were not in both sites with two exceptions. In India there was a lower rate of facility deliveries (94.3% versus 98.3%; *p* = 0.006) among babies who were not assessed at 18 months compared to those who were, and in Pakistan there was a percentage of mothers with no education (18.6% versus 13.4%, *p* = 0.005).

**Table 2:** Comparability at recruitment between trial arms for participants with an 18 month CDA.

Indicator	Control	Intervention	I-C Difference (95% CI)	*p*
**(A)** India				
Number with 18-month assessment	736	707		
% Mothers no education	6.3% (46)	5.9% (42)	−0.34% (−3.10, 2.42)	0.809
Maternal education level attained*: mean (sd)	2.91 (1.44)	2.99 (1.46)	0.06, (−0.26, 0.38)	0.717
% scheduled/backward caste/tribe (*n*)	58% (427)	62.4% (441)	6.67% (−16.23, 29.57)	0.568
% poorest (lowest 2 quintiles) (*n*)	42.7% (314)	41.3% (292)	−0.49% (−12.63, 11.65)	0.937
% Male (*n*)	54.6% (402)	52.3% (370)	−2.28% (−7.60, 3.04)	0.401
% Twins/Triplets (*n*)	1.4% (10)	1.4% (10)	0.00% (−1.08, 1.08)	0.994
% Delivered in facility (*n*)	98.1% (722)	98.4% (696)	0.35% (−1.00, 1.69)	0.614
Mean age of mother at delivery (sd)	22.1 (3.7)	22.5 (3.8)	0.360 (−0.232, 0.952)	0.233
Mean age of child at assessment (sd)	18.4 (0.28)	18.3 (0.32)	−0.03 (−0.17, 0.11)	0.676
Mean SES score (sd)	−0.07 (2.66)	−0.06 (2.69)	−0.019 (−0.710, 0.673)	0.957
**(B)** Pakistan				
Number with BSID-III assessment (18 m)	505^1^	510		
% Mothers no education	12.3% (62)	14.1% (72)	1.66% (−4.10, 7.43)	0.572
Maternal education – number of years: mean (sd)	7.9 (4.18)	7.8 (4.38)	−0.15 (−0.90, 0.60)	0.702
% poorest (lowest 2 quintiles) (*n*)	39.6% (200)	42.2% (215)	2.2% (−11.16, 15.62)	0.744
% Male (*n*)	53.9% (272)	50.6% (258)	−3.28% (−9.94, 3.39)	0.336
% Twins/Triplets (*n*)*	1.2% (6)	1.2% (10)	0.77% (−0.76, 2.30)	0.328
% Delivered in facility (*n*)	91.7% (463)	89.2% (455)	−2.41% (−6.45, 1.63)	0.242
Mean age of mother at delivery (sd)	27.0 (4.50)	26.6 (4.52)	0.42 (−1.31, 0.98)	0.134
Mean age of child at assessment (sd)	18.7 (0.30)	18.7 (0.30)	0.01 (−0.08, 0.11)	0.791
Mean SES score (sd)	0.004 (2.326)	−0.093 (2.492)	−0.092 (−0.74, 0.56)	0.781

^1^Excludes 1 child with missing information. *Only one twin/triplet assessed at 18 m.

### Impact of SPRING

The SPRING intervention failed to have an impact in either India or Pakistan on child development outcomes (Table 3) or growth (Table 4) at 18 months of age, or on the HOME-IT scores of the quality of the home environment for child development at 12 months of age (Table 5).

**Table 3:** Effect of the SPRING intervention on early child development: assessed at 18 months of age using BSID-III.

Outcomes	Mean scores (SD)			Intervention – Control (I-C) difference (95% CI)	Effect size (95% CI)	*p* value
	Overall	Control	Intervention			
**India**						
**No. of children**	1,443	736	707			
BSID-III: composite psychomotor score	94.5 (9.79)	94.4 (9.87)	94.5 (9.70)	0.06 (−1.79, 1.92)	0.00 (−0.10, 0.11)	0.946
BSID-III: composite cognitive score	92.6 (10.69)	92.9 (10.48)	92.3 (10.90)	−0.62 (−2.66, 1.43)	−0.03 (−0.13, 0.07)	0.554
BSID-III: composite language score	90.5 (14.31)	91.0 (14.52)	90.0 (14.07)	−0.96 (−4.54, 2.62)	−0.03 (−0.13, 0.08)	0.601
**Pakistan**						
**No. of children**	1,016	506	510			
BSID-III: composite psychomotor score	98.1 (12.05)	98.7 (11.65)	97.5 (12.41)	−1.18 (−2.66, 0.30)	−0.10 (−0.22, 0.02)	0.118
BSID-III: composite cognitive score	90.2 (9.65)	90.3 (9.75)	90.1 (9.56)	−0.16 (−1.86, 1.54)	−0.01 (−0.13, 0.11)	0.852
BSID-III: composite language score	93.5 (12.45)	93.9 (12.73)	93.0 (12.17)	−0.90 (−2.72, 0.93)	−0.06 (−0.18, 0.06)	0.336

**Table 4:** Effect of the SPRING intervention on child growth, measured at 18 months of age.

	Mean *Z*-Scores (SD)			I-C difference (95% CI)	Effect size (95% CI)	*p-*value
	Overall	Control	Intervention			
**India**						
**No. of children**	1,443	736	707			
Height for age *z*-score	−1.79 (1.08)	−1.80 (1.12)	−1.77 (1.04)	0.03 (−0.14, 0.21)	0.02 (−0.08, 0.12)	0.710
Weight for age *z*-score	−1.36 (1.01)	−1.37 (1.05)	−1.36 (0.97)	0.00 (−0.15, 0.15)	0.00 (−0.10, 0.10)	0.997
Weight for height *z*-score	−0.69 (0.93)	−0.68 (0.94)	−0.70 (0.92)	−0.02 (−0.13, 0.08)	−0.02 (−0.14, 0.08)	0.677
**Pakistan**						
**No. of children**	1012^1^	505	507			
Height for age *z*-score	−1.16 (1.23)	−1.14 (1.24)	−1.19 (1.21)	−0.05 (−0.29, 0.18)	−0.03 (−0.15, 0.09)	0.648
Weight for age *z*-score	−1.08 (1.13)	−1.11 (1.13)	−1.05 (1.13)	0.06 (−0.12, 0.24)	0.04 (−0.08, 0.17)	0.511
Weight for height *z*-score	−0.72 (1.15)	−0.78 (1.15)	−0.66 (1.16)	0.12 (−0.08, 0.31)	0.08 (−0.05, 0.19)	0.244

^1^Height and weight measurements not available for 4 children with BSID-III assessment; 1 in the control group and 3 in the intervention group.

**Table 5:** Effect of the SPRING intervention on intermediate outcomes assessed at 12 months of age: HOME-IT Mean Scores.

Total and sub-scale scores (no. of items involved)	Mean HOME-IT scores (SD)			I-C difference (95% CI)	Effect size (95% CI)	*p-*value
	Overall	Control	Intervention			
**India**						
**No. of children**	1,303	658	645			
Total (45)	31.6 (4.12)	31.2 (4.30)	32.1 (3.87)	0.85 (−0.35, 2.04)	0.08 (−0.03, 0.19)	0.164
Responsivity (11)	9.2 (1.62)	9.1 (1.72)	9.3 (1.50)	0.23 (−0.27, 0.74)	0.05 (−0.06, 0.16)	0.361
Acceptance of child’s behaviour (8)	6.8 (0.86)	6.8 (0.88)	6.7 (0.84)	−0.05 (−0.22, 0.11)	−0.03 (−0.15, 0.08)	0.521
Organisation of the environment (6)	4.7 (1.02)	4.6 (1.04)	4.8 (0.99)	0.19 (−0.01, 0.39)	0.11 (−0.01, 0.22)	0.061
Learning materials (9)	4.9 (1.78)	4.7 (1.81)	5.2 (1.72)	0.43 (−0.05, 0.91)	0.10 (−0.01, 0.21)	0.078
Parental involvement (6)	4.0 (0.86)	3.9 (0.88)	4.0 (0.84)	0.11 (−0.10, 0.32)	0.06 (−0.05, 0.16)	0.304
Variety (5)	2.1 (0.88)	2.1 (0.87)	2.1 (0.88)	−0.06 (−0.18, 0.06)	−0.06 (−0.17, 0.06)	0.345
**Pakistan**						
**No. of children**	1232^1^	622	610			
Total (45)	27.3 (5.06)	26.8 (4.91)	27.7 (5.19)	0.92 (−0.89,2.74)	0.06 (−0.05, 0.17)	0.320
Responsivity (11)	6.7 (1.94)	6.6 (1.93)	6.8 (1.94)	0.27 (−0.43,0.96)	0.04 (−0.07, 0.16)	0.448
Acceptance of child’s behaviour (8)	6.5 (1.19)	6.5 (1.05)	6.5 (1.32)	−0.06 (−0.30,0.18)	−0.03 (−0.14, 0.09)	0.638
Organisation of the environment (6)	3.5 (1.26)	3.3 (1.24)	3.6 (1.27)	0.27 (0.08,0.46)	0.15 (0.05, 0.26)	0.005
Learning materials (9)	3.9 (2.27)	3.8 (2.26)	4.1 (2.28)	0.27 (−0.38,0.91)	0.05 (−0.07, 0.16)	0.420
Parental involvement (6)	4.0 (0.90)	3.9 (0.93)	4.0 (0.87)	0.12 (−0.20,0.44)	0.04 (−0.07, 0.16)	0.454
Variety (5)	2.7 (0.72)	2.7 (0.76)	2.7 (0.69)	0.05 (−0.06,0.17)	0.05 (−0.06, 0.16)	0.361

^1^HOME-IT inventory not completed for 2 children with 12 m CDA; 1 in the control group & 1 in the intervention group.

However, it did appear to have had some impact on reported feeding practices at 12 months of age (Table 6). The percentage of children in the SPRING intervention group who were receiving diets at 12 months of age that met the WHO minimum acceptable criteria compared to the percentage of those in the control group was 35% higher in India (95% CI: 4–75%, *p* = 0.023) and 45% higher in Pakistan (95% CI: 15–83%, *p* = 0.002). In both countries this appears to be largely due to an increase in the percentage of babies receiving 4 or more food groups. In Pakistan but not India, there was also a modest increase increase (*p* = 0.016) in the percentage receiving the recommended number of meals. However, the majority of children in the intervention group were still not receiving adequate diets; this was 68.5% in India and 57.7% in Pakistan.

**Table 6:** Effect of the SPRING intervention on intermediate outcomes assessed at 12 months of age: complementary feeding (*CF*) practices.

	% Infants (n/N)			RR (95% CI)	*p-*value
	Overall	Control	Intervention		
**India**					
**No. of children**	1,303^1^	658	645		
Infants meeting WHO criteria for minimum acceptable diet at 12 months of age	27.5% (549)	23.7% (239)	31.5% (310)	1.35 (1.04, 1.75)	0.023
Receiving recommended number of meals	75.8% (1513)	73.9% (746)	77.9% (767)	1.06 (0.97, 1.15)	0.194
Receiving 4 or more food groups	34.4% (686)	30.4% (307)	38.5% (379)	1.27 (1.05, 1.55)	0.016
**Pakistan**					
**No. of children**	1,197^2^	604	593		
Infants meeting WHO criteria for minimum acceptable diet at 12 months of age	35.8% (429)	29.5% (178)	42.3% (251)	1.45 (1.15, 1.83)	0.002
Receiving recommended number of meals	87.4% (1046)	85.1% (514)	89.7% (532)	1.05 (1.01, 1.10)	0.016
Receiving 4 or more food groups	44.9% (537)	38.7% (234)	51.1% (303)	1.33 (1.08, 1.65)	0.008

^1^In India, 12 m feeding questionnaire was administered to all mothers who had a 12 m assessment and not just those whose children were recruited for the 18 m CDA, giving an additional 716 children, 365 in the control group and 351 children in the intervention group. ^2^In Pakistan, feeding questionnaire was not completed for 37 children with 12 m assessment: 19 in the control group and 18 in the intervention group.

There were also major gaps in recommended feeding practises in the first 6 months of life (Table 7). Only 50.4% of babies in India were breastfed within the recommended hour of birth and only 12% in Pakistan; the rates were comparable in intervention and control groups. The exclusive breastfeeding rates in the fifth and sixth months of life (age 4–5 m) were higher in the intervention groups in both countries but these were still below 50%; the majority the majority of babies had stopped exclusive breastfeeding before the fifth month. In India, the intervention successfully tackled the problem of late weaning, highlighted as a major problem during the formative research – the percentage of infants receiving solids/semisolid food at the recommended age of 6 months was considerably higher in the intervention group than the control group, 51.9% compared to 28.8%, an estimated increase of 79% (95% CI 37–134%, *p* < 0.001), although almost half the children were still not receiving food at this age. There was no similar improvement in Pakistan where the rates were much higher in both groups with the majority (70.7%) of infants receiving solids/semisolid food at 6 months.

**Table 7:** Effect of the SPRING intervention on intermediate outcomes collected during surveillance visits: breastfeeding and start of weaning.

	% Infants (n/N)			RR (95% CI)	*p-*value
	Overall	Control	Intervention		
India^1,3^					
Babies breastfed within an hour of birth	50.4% (2,535/5031)	50.5% (1,269/2513)	50.3% (1,266/2518)	1.00 (0.88–1.14)	0.977
Exclusively breastfed in period 4-5 m^4^	40.1% (1753/4373)	32.4% (722/2225)	48.0% (1,031/2148)	2.21 (0.90–5.44)	0.084
Infants receiving solids/semisolid food at 6 months of age^5^	39.9% (849/2126)	28.8% (317/1100)	51.9% (532/1026)	1.79 (1.37–2.34)	<0.001
Pakistan^2,3^					
Babies breastfed within an hour of birth	12.0% (581/4831)	14.1% (326/2315)	10.1% (255/2516)	0.69 (0.36, 1.29)	0.243
Exclusively breastfed in period 4–5 m^3^	34.8% (1,194/3436)	30.7% (509/1656)	38.5% (685/1780)	1.29 (1.05, 1.59)	0.015
Infants receiving solids/semisolid food at 6 months of age 5	70.7% (1,116/1579)	73.2% (567/773)	68.1% (549/806)	0.93 (0.84, 1.03)	0.143

^1^India: 5117 babies were born on or after 18 June 2015 and enrolled in the surveillance system up to 01 July 2017. Breastfeeding data were collected for 5,032 babies; data on breastfeeding initiation was missing for one of these. This is less than the total number as breastfeeding data were not collected from children who had died by the time the birth was identified by the surveillance fieldworker. ^2^Pakistan: babies were born after 2 May 2014 and enrolled in the surveillance system up to 30 April 2016. Breastfeeding data were collected for 4,831 babies. ^3^The number of babies with breastfeeding data is less than the total number of babies as breastfeeding data were not collected from children who had died by the time the birth was identified by the surveillance fieldworker. ^4^Some babies contributed more than one entry to this age period, and some contributed none depending on when their surveillance visits occurred. All analyses have been adjusted for these repeated measures. ^5^Based on infants who had a surveillance visit when they were 6 months of age.

Intra-cluster coefficient for outcome measures are presented in Table 8. They are considerably lower than the assumed values for the primary outcomes of 0.05 for the BSID scores and 0.35 for HAZ used in the sample size calculations, with the exception of the composite language score in India which had an ICC of 0.78.

**Table 8:** SPRING outcomes: intraclass correlation coefficients (ICCs).

Outcome	India	Pakistan
BSID-III: composite psychomotor score	0.0377481	0.0000000
BSID-III: composite cognitive score	0.0392897	0.0190526
BSID-III: composite language score	0.0787696	0.0083743
Height for age *z*-score	0.0245728	0.0251728
Weight for age *z*-score	0.0159574	0. 0123319
Weight for height *z*-score	0.0034404	0.0190417
HOME-IT Score	0.1198204	0.1523026
% Infants meeting WHO criteria for minimum acceptable diet	0.0492589	0.0535477

#### Robustness checks

Additional analyses adjusted for month of birth, socio-economic status (SES) score and cluster level baseline factors (% stunting among children aged 18–30 months, % of mothers with no education among mothers with children aged less than 5 years and % of youngest children less than 5 years of age delivered in a facility) did not materially change the results for any outcome. Similarly, using randomisation inference rather than random effects regression provided estimated results which were almost identical.

### Economic evaluation

In Pakistan, the total incremental cost from the provider perspective of SPRING home visits over the duration of the programme was INT$ 654,164, and the average annual cost of delivery was INT$ 167,021. In India, the total provider cost of SPRING home visits was INT$ 727,717, of which INT$ 283,994 was attributed to the salaries of the project-appointed KWs. The average annual cost of delivery in India was INT$ 291,887.

## Discussion

The SPRING programme has several strengths. It evaluated a culturally appropriate intervention which was based on existing global guidelines and designed using best practise in relation to formative research, counselling practises and supportive supervision (24–27, 46). SPRING was also designed from the outset to test the delivery using two different feasible delivery models for scale-up that could be taken up by governments. This contrasts with most trials to date which have provided evidence that parent-focussed face to face interventions promoting nurturing care can be effective in improving child development outcomes (15–17). These have mostly tested interventions delivered to at-risk or small populations and/or with considerable levels of input to ensure quality of the intervention delivery. These high intensity interventions are neither affordable nor feasible at scale in most low- and middle-income countries, where the need is greatest. In Pakistan, SPRING worked in close partnership with the long standing LHW programme, to integrate the SPRING content and approach into the monthly home visits provided by the LHWS. In India, homes visits were implemented by a civil society organisation (NGO) that trained a new cadre of community agents to work alongside the existing services provided by ASHAs and Anganwadi workers.

SPRING was evaluated through rigorously designed cluster randomised controlled trials in both settings accompanied by detailed economic and process evaluations. The trials had high follow-up rates covering large whole-population samples and culturally adapted outcome measures. These trials show that the SPRING intervention failed to have any impact on child development or growth in either India or Pakistan. It did appear to have some impact on reported feeding practises (both breast- and complementary-feeding) in both countries, but this did not translate into change in growth outcomes. It is important to note that the majority of children in the intervention arm continued to receive inadequate diets: 68.5% in India and 57.7% in Pakistan, and late weaning remained a major problem in India. It is also important to note that the criteria for the WHO minimum acceptable diet, which are based on the number of meals and the diversity of the diet, do not guarantee that the baby is necessarily receiving an adequate amount of food.

This lack of impact on early child development in both settings in the SPRING trial is in contrast to the impact seen in the smaller scale effectiveness trials (17) that informed the WHO guideline on improving early child development (18). This evidence included 18 trials with combined caregiving and nutrition interventions (18), including the Pakistan Early Child Development Study (PEDS) (47). The PEDS study demonstrated, as a proof of principle, that ECD interventions can be effectively delivered through the LHW programme; significant impacts were found for a range of ECD outcomes but not for growth.

The detailed process evaluation reported in a companion paper (22) explores what could have caused this lack of impact in the SPRING trial, and draws lessons that can be learned for future scale-up. Implementation was sub-optimal in both settings. Problems due to political and logistical barriers, workforce constraints, low supervision and a lack of early child development skills development impacted the quality of home visits when using the existing LHWs in Pakistan. The intervention specific KWs in India conducted higher quality visits, but coverage was low in part due to employing new workers and an empowerment approach to visit scheduling. Coaching caregivers on skills, a key intervention element, was sub-optimal in both sites, and is likely to have contributed to caregiver perceptions that the intervention content was not new and was focused on play activities rather than interaction and responsivity. In both sites caregiver time pressures was a key reason for low uptake among families who received visits. The embedding periods of 2–3 months were also not long enough to ensure all operational aspects were fully functioning.

The absence of an observable effect of the SPRING intervention in either trial on the main outcomes although disappointing has important policy implications for achieving feasible and effective scale up as countries increase their efforts to improve early child development, nutrition and growth. The findings emphasise that impacts achieved in efficacy or proof of principle trials may not be achieved when interventions are delivered routinely at scale. The findings are consistent with the experience of scaling up Crianca Feliz in Brazil which found no impact on a range of ECD outcomes and reported that rapid scale up was a barrier to achieving quality and consistency (48); this is an important example that coverage should not be prioritised at the expense of quality (49). A programme review of 10 country experiences of scaling up CW postnatal home visits also demonstrates the difficulty of achieving high coverage at scale, with most countries achieving less than 10% coverage and none achieving more than 20% coverage (50); the magnitude of this challenge should not be overlooked.

Integrating additional tasks into the workload of already overloaded and stressed CWs can be challenging in low-capacity contexts or weak health systems (51–54). Simply adding a child development and nutrition component is unlikely to be successful without additional resources and re-organisation of the goals of the existing community-based intervention. The India ‘NGO delivery model’ with project specific CWs is likely to mirror the way expansions will occur in many low- or middle-income countries, at least in the short term, as few have the established infrastructure, systems and experience with community-based workers, such as LHWs, into which the promotion of early child development and nutrition can be integrated. This model does not guarantee adequate coverage, however.

Programmes will need strategies to maximise both coverage and quality of intervention delivery, including having a clear strategy for scheduling visits, monitoring coverage and other process indicators, identifying and managing poor performers, and developing feedback loops for course correction. Rapid scale will therefore require careful attention to the establishment of strong administrative and management systems to support its implementation.

## Data availability statement

The datasets presented in this study can be found in online repositories. The names of the repository/repositories and accession number(s) can be found at: World Bank International Household Survey Network (IHSN) microdata catalogue (https://catalog.ihsn.org/catalog/7952/study-description). LSHTM Data Compass data repository (https://doi.org/10.17037/DATA.00003124).

## Ethics statement

The studies involving human participants were reviewed and approved by London School of Hygiene & Tropical Medicine (LSHTM) Research Ethics Committee (UK), the Ethical Review Committee at the Human Development Research Foundation (Pakistan) and the Sangath Institutional Review board (India). Approval was also granted by the Indian Council of Medical Research’s Health Ministry Screening Committee (HMSC). Written informed consent to participate in this study was provided by the participants’ legal guardian/next of kin.

## Author contributions

BRK, SiS, RR, RL, SB, BA, NB, BK, JS, ZH, and AR designed the cluster randomised trials and data collection instruments. ZH, SZ, GD, AH, SiS, RL, BRK, and AR designed the content of the SPRING home visits intervention. SiS, RR, SA, DK, DV, and KS were responsible for the surveillance system and outcome data collection. LG, SP, RR, SB, SiS and SA designed and managed the data management system and scheduling of the surveillance visits and 12 and 18 month assessments. LG was the independent trial statistician responsible for the restricted randomisation. SeS was the independent trial statistician responsible for all the analyses presented in this manuscript. BRK wrote the first draft of the manuscript which was then reviewed by all authors. All authors contributed to the article and approved the submitted version.

## Funding

SPRING was funded by a Wellcome Trust Programme Grant (Award No: 093615). As well as the development of the SPRING intervention and its evaluation through cluster randomised controlled trials, the grant also covered implementation costs in India, where SPRING was delivered by project appointed KWs. In Pakistan, salaries of the LHWs were covered by the LHW programme, with the programme grant covering additional costs relating to training, monitoring, and enhanced supervision. Supplementary funding was received from the World Bank Strategic Impact Evaluation Fund (SIEF; Contract No: 7180224) to enable additional data collection on intermediate variables and a detailed process and implementation evaluation in order to develop a more detailed understanding of the pathways through which any impacts occurred. The funders had no role in the trial design, data collection, data analysis, data interpretation, or writing of the report apart from the recommendation by SIEF to include a range of robustness checks. The corresponding author had full access to all the data in the study and had final responsibility for the decision to submit for publication.

## Conflict of interest

The authors declare that the research was conducted in the absence of any commercial or financial relationships that could be construed as a potential conflict of interest.

## Publisher’s note

All claims expressed in this article are solely those of the authors and do not necessarily represent those of their affiliated organizations, or those of the publisher, the editors and the reviewers. Any product that may be evaluated in this article, or claim that may be made by its manufacturer, is not guaranteed or endorsed by the publisher.
